# Radiation-Induced Sharpening in Cr-Coated Zirconium Alloy

**DOI:** 10.3390/ma15062322

**Published:** 2022-03-21

**Authors:** Joël Ribis, Alexia Wu, Raphaëlle Guillou, Jean-Christophe Brachet, Cédric Baumier, Aurélie Gentils, Marie Loyer-Prost

**Affiliations:** 1 Université Paris-Saclay, CEA, Service de Recherches Métallurgiques Appliquées, 91191 Gif-sur-Yvette, France; wu.alexia@gmail.com (A.W.); raphaelle.guillou@cea.fr (R.G.); jean-christophe.brachet@cea.fr (J.-C.B.); 2 Université Paris-Saclay, CNRS/IN2P3, IJCLab, 91405 Orsay, France; cedric.baumier@in2p3.fr (C.B.); aurelie.gentils@ijclab.in2p3.fr (A.G.); 3 Université Paris-Saclay, CEA, Service de Recherches de Métallurgies Physiques, 91191 Gif-sur-Yvette, France; marie.loyer-prost@cea.fr

**Keywords:** Cr-coated zirconium alloys, irradiation, dissolution, sharpening, transmission electron microscopy

## Abstract

To improve the safety of nuclear power plants, a Cr protective layer is deposited on zirconium alloys to enhance oxidation resistance of the nuclear fuel cladding during both in-service and hypothetical accidental transients at High Temperature (HT) in Light Water Reactors. The formation of the Cr_2_O_3_ film on the coating surface considerably helps in reducing the oxidation kinetics of the zirconium alloy, especially during hypothetic Loss of Coolant Accident (LOCA). However, if the Cr coating is successful to increase the oxidation resistance at HT of the zirconium substrate, for in-service conditions, under neutron irradiation, Cr desquamation has to be avoided to guarantee a safe use of the Cr-coated zirconium alloys. Therefore, the adhesion properties have to be maintained despite the structural defects created by sustained neutron irradiation in the reactor environment. This paper proposes to study the behavior of the Zircaloy-Cr interface of a first generation Cr-coated material during a specific in situ ion irradiation. As deposited, the Cr-coated sample presents a f.c.c. C15 Laves-type intermetallic phase at the interface with off-stoichiometric composition. After irradiation and for the specific conditions applied, this interfacial phase has significantly dissolved. Energy Dispersion Spectroscopy revealed that the dissolution was accompanied by a counterintuitive “sharpening” effect.

## 1. Introduction

In a Light Water Reactor (LWR), zirconium-based alloys are commonly used for nuclear fuel claddings. However, during LOCA (Loss of Coolant Accident) transients, these fuel claddings are exposed to oxidation by steam at a high temperature. The result is a severe embrittlement of the zirconium alloy. To avoid such a scenario, the oxidation resistance of the nuclear fuel claddings can be improved through the concepts of Enhanced Accident Tolerant Fuel (EATF) [1]. Among the different evolutive (i.e., short-term) EATF concepts studied so far, Cr-coated Zr-based claddings appear to be one of the most promising [2,3,4,5]. In doing so, metallic chromium-based coatings are deposited on zirconium cladding tubes using different processes. This protective layer aims to increase the zirconium resistance to steam oxidation owing to the compact Cr_2_O_3_ film, which forms on the coating surface at high temperatures. Furthermore, the adhesion property of the Cr coating is excellent since no cracks or peel offs were observed after both ring compression and tensile tests [6]. After the Physical Vapor Deposition (PVD) process, the microstructure of the Cr coating generally consists of columnar-shaped grains, while small equiaxed grains can be observed close to the Zr/Cr interface. In the case of a first generation Cr-coated Zircaloy-4 type substrate, TEM exams also reveal that interdiffusion between Cr and Zr atoms leads to the formation of a C14-C15 polystructured Laves phase at the interface [7,8]. If the oxidation resistance properties of the Cr-coated zirconium alloy are very promising [5], one has to keep in mind that this coated zirconium alloy will be subjected to neutron irradiation once in the reactor. Therefore, the radiation-resistance of such a system must also be assessed and guaranteed.

The response under irradiation of the Cr layer was examined recently by Wang et al. [9], who proposed to study the deformation behavior of irradiated Cr coatings under nanoindentation. The coatings were irradiated with 5 MeV Xe^20+^ ions at room temperature up to a dose of 0.26 dpa at the depth of 600 nm. The authors found that the irradiation caused a significant increase in the coating hardness and a decrease in the indentation modulus. The authors also reported Cr column binding in the indented irradiated area. Kuprin et al. [10] did not observe Cr column binding but a significant grain growth after ion irradiation in the range of 300–500 °C. They also observed the formation of cavities and reported that the swelling caused by void formation is maximum at 500 °C. Gong et al. [11] also observed voids and brought new precisions on their formation by showing that the void size and density depend on the Cr grain texture: void size decreases while void density increases with increasing the (110)-oriented crystallinity. Although various authors reported voids, they are not the only defects observed under irradiation. Jiang et al. [12] observed dislocation loops after irradiation at 10, 25, and 50 dpa at 400 °C, which are responsible for the hardening of the irradiated Cr coating. In addition to the irradiated Cr layer, some authors have also been interested in the stability of the interface between the zirconium substrate and the chromium layer. Wu et al. [8] showed that after ion irradiation up to 10 dpa, the interface region remained stable and only slight Fe segregation was reported. This segregation comes from the dissolution of the neighbor Laves phases in the zirconium substrate and results in the stabilization of the C14 Laves phase over the C15 one. Wu et al. [8] also concluded that atom row matching is still preserved after ion irradiation between Cr and Laves phases and between Zr and Laves phases. However, this last work concerns only the stationary state, raising some questions as to the existence of a transient regime occurring before the stabilization of the C14 phase. Therefore, to complement and reinforce the knowledge on the radiation response of the Cr-coated zirconium alloy, the present paper proposes to study, by means of in situ ion irradiation, the stability of the interfacial Laves phase-type that has formed in the Zr–Cr interface region. It is worth noting that only ion irradiation has been used in this paper, therefore the results obtained can be partially used for the interpretation of the phenomenon that may occur under neutron irradiation. 

## 2. Materials and Methods

The base material is a first-generation 1.2 mm thick low-tin Zircaloy-4 sheet coated with Cr by a PVD process. The chemical composition of the Zy-4 alloy is presented in Table 1. After deposition, the typical Cr-coating thickness reached an average value of 0.6 µm. One has to keep in mind that this Cr-coated sample corresponds to a first generation Cr-coated Zircaloy-4 sheets and is not fully representative of the last generation Cr-coated zirconium alloys developed in the framework of CEA-EDF-FRAMATOME collaborative program [13].

Chemical analyses and microstructure observations were performed by means of conventional transmission electron microscopy (TEM), as well as Energy Filtered and High-Resolution TEM. The observations were conducted on a dual aberration-corrected JEOL-neoARM microscope operating at 200 kV (JEOL LTD, Tokyo, Japan), equipped with double-Centurio EDS (Energy Dispersion Spectroscopy) detectors (JEOL LTD, Tokyo, Japan), with GIF-Continuum (Gatan INC, Pleasanton, CA, USA) and on a JEOL-2010F microscope operating at 200 kV and equipped with an Oxford (OXFORD INSTRUMENT Group, Tubney Wood, UK) EDS detector. Focused Ion Beam (FIB) technique was used to extract a standard cross-section preparation from both virgin and irradiated Cr-coated Zircaloy-4 samples with FEI Helios SEM/FIB dual-beam microscope (Thermo Fisher Scientific, Waltham, MA, USA).

In situ ion irradiations were conducted at the JANNuS-Orsay facility [14] at IJClab equipped with a FEI Tecnai G^2^ 20 TEM (Thermo Fisher Scientific, Waltham, MA, USA) operating at 200 kV formerly equipped with EDAX (EDAX INC, Mahwah, NJ, USA) EDS detectors and coupled with the ARAMIS 2 MV ion accelerator. Irradiations were performed at 400 °C. The irradiations were conducted using 4 MeV Au^2+^ ions with a flux of 2.8 × 10^11^ ions.cm^−2^s^−1^. The final fluence was 4.9 × 10^15^ ions.cm^−2^ and the damage rate was about 1.4 × 10^−3^ dpa.s^−1^. In situ ion irradiation were performed on FIB lamella samples tilted by 32° with respect to the incoming beamline. It is worth noting that both Zr and Cr regions are irradiated at the same time. In in situ conditions, owing to focus disruption due to temperature application, TEM images were acquired before and after irradiation with correct focus conditions.

**Figure 1:**
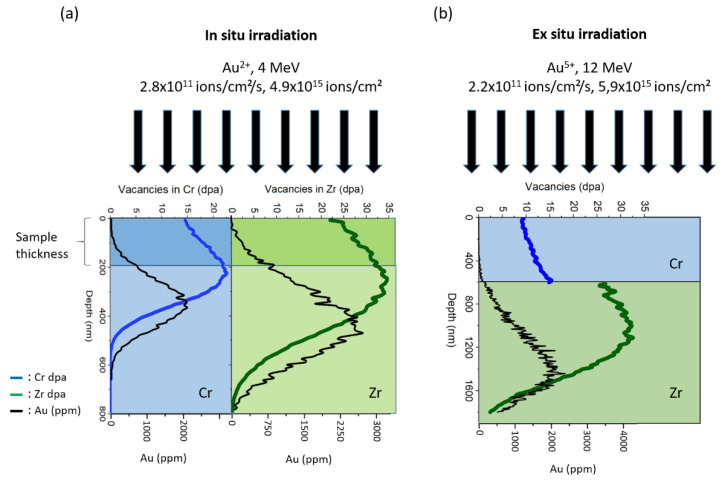
Depth profile of displacement damage under gold ion irradiation in: (**a**) in situ irradiation condition, (**b**) ex situ irradiation condition.

The depth profile of the irradiation damage was calculated using Kinchin-Pease mode from the Iradina software (CEA, Saclay, France) [15] with 40 eV displacement energy. The corresponding irradiation damage profile is presented in Figure 1a.

The ex situ ion irradiations were conducted at the JANNuS-Saclay facility [14] using 12 MeV Au^5+^ ions with a flux of 2.2 × 10^11^ ions.cm^−2^s^−1^. Specimens were bulk samples maintained at a nominal temperature of 400 °C and was tilted by 15° with respect to the incoming ion beamline. The final fluence was 5.9 × 10^15^ ions.cm^−2^ and the damage rate was about 8.1 × 10^−4^ dpa.s^−1^. The total damage at the interface reached an average value of 20 dpa. In this irradiation experiment, contrariwise to the in situ irradiation, Zr and Cr were not irradiated at the same time, but Au^5+^ ions first penetrated the Cr region and then stopped in the Zr region. 

The depth profile of displacement damage for ex situ irradiation is presented in Figure 1b for both Zr and Cr regions. It was calculated using Kinchin-Pease mode from the Iradina software [15] with 40 eV displacement energy. About 25 and 15 dpa were respectively reached in Zr and Cr regions over the first 100 nm, which corresponds to the FIB lamella thickness.

It is well known that PVD process may induce some internal stresses within the Cr-coating. To quantify them, internal stress levels in unirradiated and irradiated chromium layer (ICDD 00-006-0694) (International Center for Diffraction Data, Newtown Square, PA, USA) were determined by in situ X-ray diffraction at room temperature under atmospheric pressure conditions. This experiment was performed on bulk samples. Determinations were made by using Ψ goniometer technique, a Bruker D8 discover with Cu-Kαl = 0.154 nm radiation, based on the (310) planes of the chromium structure (0.912 d0). The Ψ angle was varied in steps of 5° between 0° and 60°. The mean stress σϕ in the plane of the chromium layer is obtained from the slope (1 + ν) σϕ/E of the linear relationship between ε and sin2Ψ, where ε is the strain, given by Δd/d, d being the interplanar spacing. The chromium layer was assumed to be isotropic with no stress gradients parallel to the surface components. The chromium Young’s modulus, E, was taken as 280 GPa at room temperature and Poisson’s ratio ν as 0.22. It must be kept in mind that the technique used is not able to determine the eventual stress gradient inside the layer. Only a mean value is obtained from the whole surface and thickness analyzed.

## 3. Results

### 3.1. Characterization of the Cr-Coated Zircaloy-4 before Irradiation

**Figure 2:**
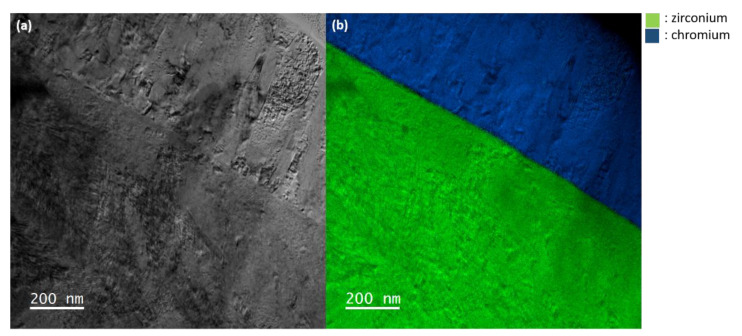
TEM images of the Cr-coated zircaloy-4: (**a**) bright-field TEM showing both the Zr and Cr regions, (**b**) energy-filtered jump ratio images obtained at the Zr M and Cr M edges.

Figure 2a is a TEM bright-field showing a general view of the FIB lamella where can be observed both the zirconium and the chromium regions. As described elsewhere [7], the Cr grains are elongated and display a columnar shape. Figure 2b is a filtered image obtained after mixing the jump ratio images acquired at the Zr M and Cr M edges. These images help to distinguish the Zr region from the Cr region. In the as-received state, the interface appears straight all along the observed area, certainly due to the low roughness of the zirconium substrate and appropriate Cr deposition conditions.

**Figure 3:**
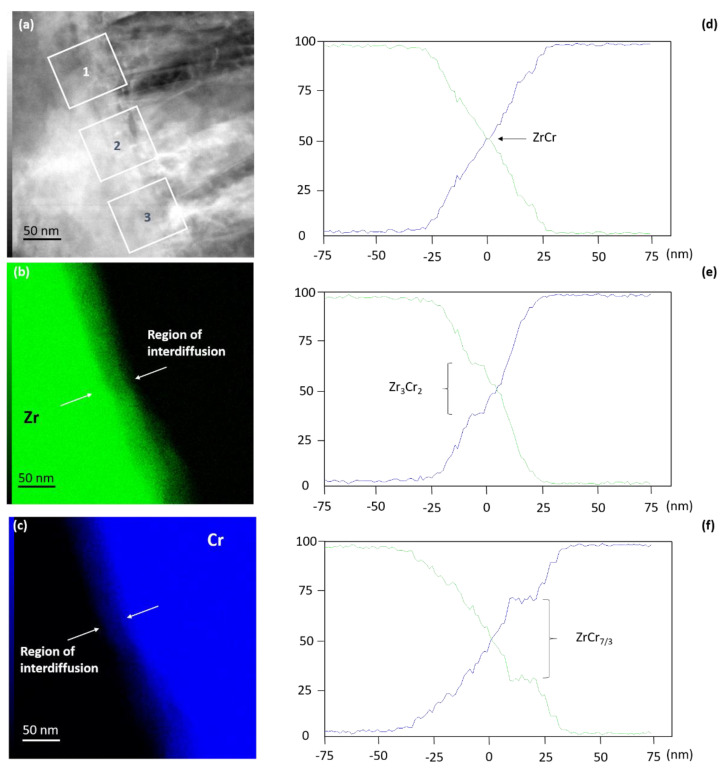
EDS analyses of the interface region: (**a**) STEM-ADF image, (**b**) EDS map obtained after collection of X-rays generated by emission from the energy-level shell Zr L, (**c**) EDS map obtained after collection of X-rays generated by emission from the energy-level shell Cr K, (**d**) Zr-Cr composition profile extracted from region labeled 1, (**e**) Zr-Cr composition profile extracted from region labeled 2, (**f**) Zr-Cr composition profile extracted from region labeled 3.

In complement to EFTEM chemical analyses, EDS acquisitions were conducted to study the Zr–Cr interface region. Figure 3a is a STEM-ADF image showing the area where X-rays were collected. Figure 3b,c are respectively Zr and Cr chemical maps obtained after the collection of X-rays generated by emission from the energy-level shells Zr L and Cr K. At a higher magnification, one can observe that the Zr–Cr interface is not abrupt but smooth owing to an apparent Zr and Cr atoms inter-diffusion. The inter-diffusion region extends on 50 nm on average and is observed all along with the studied region. Concentration profiles were extracted from three distinct areas, as depicted in Figure 3a. In the upper part of the interface, labeled 1 in Figure 3a, the Zr concentration profile as well as the Cr concentration profile appear to vary linearly from 0% to 100% across the interface region (Figure 3d). However, in the concentration profile presented in Figure 3e and extracted from the region labeled 2, a stoichiometric or near-stoichiometric phase has formed in this region, explaining why the Zr, as well as the Cr concentrations, are constant on a few nanometers. The stoichiometry of the phase has been identified to correspond to Zr_3_Cr_2_, which does not correspond to an equilibrium phase. Finally, in the lower part of the interface region (Figure 3f), labeled 3 in Figure 3a, another plateau appears, still related to a phase formation. The stoichiometry of the phase has been identified as ZrCr_7/3_, which is close to the ZrCr_2_ equilibrium phase. Therefore, one can conclude that in the interface region, the Zr and Cr atoms inter-diffusion is conducted to phase formation with probable stoichiometry deviation. Hereafter, the interface phase is denominated as Zr_x_Cr_y_. 

**Figure 4:**
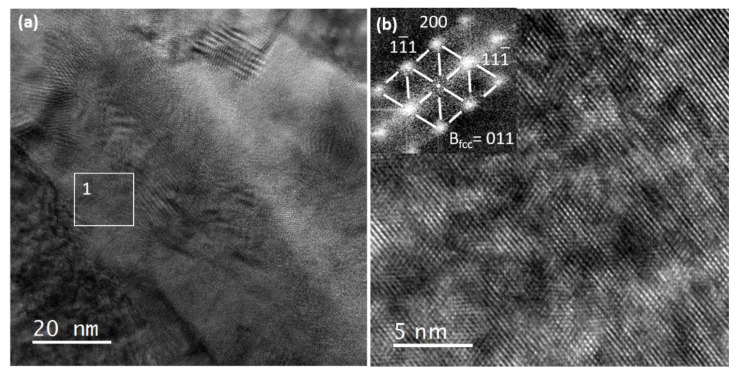
High-resolution TEM of the interface region: (**a**) bright-field TEM of the interface phase, (**b**) high-resolution image of the interface phase with corresponding Fast Fourier Transform (F.F.T.).

Since EDS acquisition did not allow to clearly identify the interfacial phase, high-resolution TEM was performed to resolve the atomic structure of the Zr_x_Cr_y_ phase. Figure 4a is a bright-field TEM image acquired in the interface region while Figure 4b is a high-resolution TEM image obtained in the region of interest labeled 1 in Figure 4a. The Fast Fourier Transform (F.F.T.) in the upper left angle of Figure 4b is clearly indexed as a 110 f.c.c. type zone axis diffraction pattern. The interplanar distance has been found to be d_200_ = 0.3 nm and d_111_ = 0.33 nm with a corresponding lattice parameter roughly equal to 0.6 nm. Therefore, the interface phase corresponds, but cannot be strictly indexed as the f.c.c. C15 ZrCr_2_ Laves phase as already observed in some other Cr-coated zircaloy-4 samples [7,8], since this last one possesses a lattice parameter equal to 0.73 nm, which is significantly higher than the lattice parameter measured in this study. Maybe, this result can be related to the deviation from the ZrCr_2_ equilibrium composition highlighted by the EDS analyses performed all along the interface region. This composition deviation can result in a slight decrease of the lattice parameter. 

### 3.2. Behavior of the Cr-Coated Zircaloy-4 under In Situ Ion Irradiation

**Figure 5:**
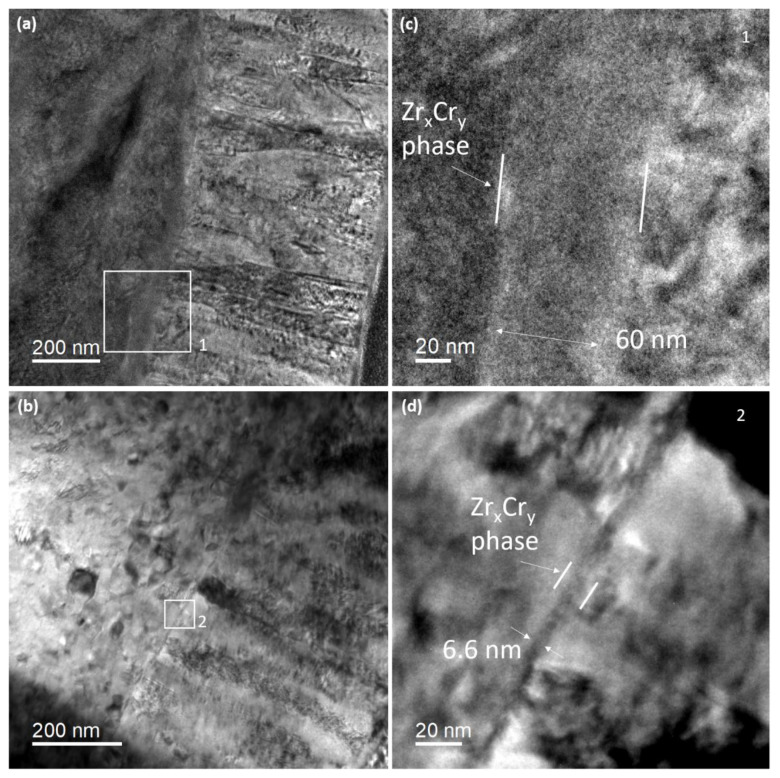
The interface region before and after irradiation: (**a**) bright field TEM image of the Zr-Cr interface, (**b**) same area after ion-irradiation, (**c**) detailed view of the interface corresponding to the white rectangle labeled 1 in (**a**), (**d**) detailed view of the interface after irradiation corresponding to the white rectangle labeled 2 in (**b**).

**Figure 6:**
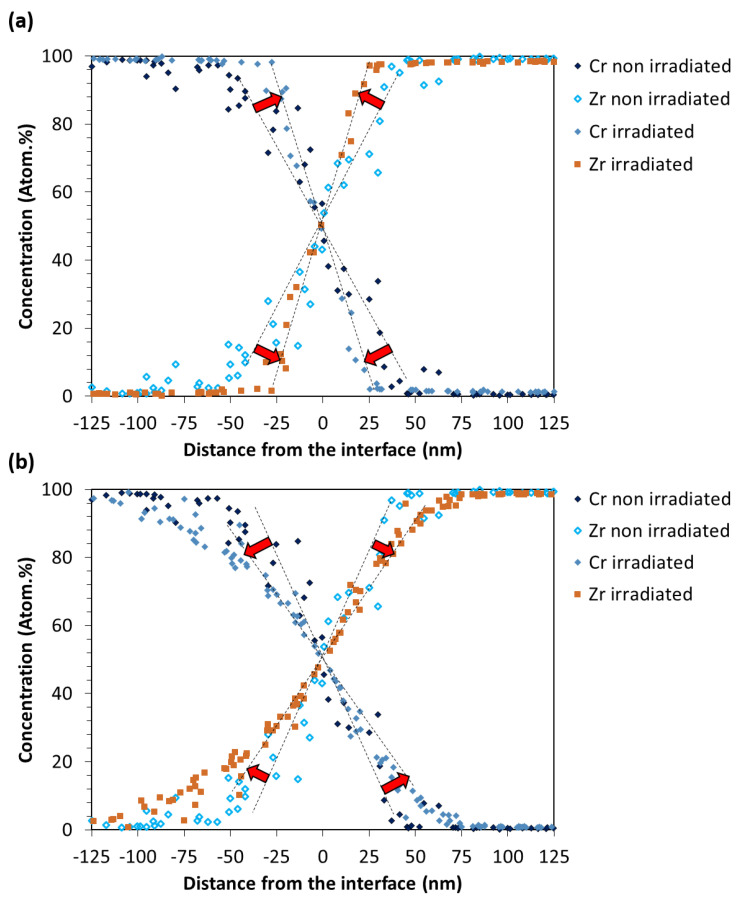
Concentration profile of Zr and Cr atoms across the interface: (**a**) before and after in situ irradiation (interface sharpening), (**b**) before and after ex situ irradiation (interface broadening).

The stability under irradiation of the Zr–Cr interface region, and more especially the stability of the Zr_x_Cr_y_ interface phase, was studied during in situ irradiation of a FIB lamella. Figure 5a presents the Cr-coated zircaloy-4 sample before irradiation. The region labeled 1 in the white rectangle shows the interface region where the change of contrast is attributed to the Zr_x_Cr_y_ interface phase, as previously observed. Before irradiation, the width of the interface phase was measured at 70 ± 10 nm (Figure 5c). Figure 6a presents the Zr-Cr composition profile across the interface before irradiation. This profile is similar to the previous ones presented in Figure 3d–f, except for the spatial resolution which is lower, explaining why the composition varies linearly from 0 to 100% for both Zr and Cr without a plateau, as observed in Figure 3e,f.

In Figure 5b, the observed region corresponds rigorously to the region presented in Figure 5a but after irradiation (10 dpa). Figure 5d proposes a detailed view of the region labeled 2 corresponding to the white rectangle in Figure 5b. By comparing Figure 5a,c with Figure 5b,d one may unambiguously conclude that the interface phase width has considerably reduced after ion-beam (irradiation) mixing. The width is estimated at roughly 6.5 ± 1.5 nm and appears constant, with few fluctuations, along with the irradiated interface. Therefore, almost 90% of the initial interface phase has “dissolved” under irradiation. Even more surprising is the comparison of the EDS composition profiles acquired across the interface before and after irradiation (Figure 6a). One could have expected that the dissolution of the interface phase would be accompanied by a broader distribution of the Zr and Cr atoms owing to the ballistic mixing. However, a counter-intuitive result occurred. The Zr-Cr chemical profile is more abrupt after irradiation than before and the interface width has reduced. It is worth noting that EDS acquisitions were performed in the same microscope with the same spectrometer. This phenomenon is called “sharpening” [16] and has already been scarcely observed in a few other interface systems, but under thermal annealing conditions [17]. It will be discussed hereafter.

### 3.3. Behavior of the Cr-Coated Zircaloy-4 under Ex Situ Ion Irradiation

In order to help the understanding of the in situ experiment, additional results were obtained on ex situ irradiated bulk samples. Figure 6b presents the chemical composition profile at the interface after ex situ ion irradiation on a bulk sample. The irradiation damage reached an average value of 20 dpa at the interface. Contrariwise, to the in situ ion irradiation, the EDS profile suggests interface broadening instead of sharpening. The interface width appears also larger, suggesting that the intermetallic interlayer phase has grown, as already reported [8] in similar irradiation conditions.

**Figure 7:**
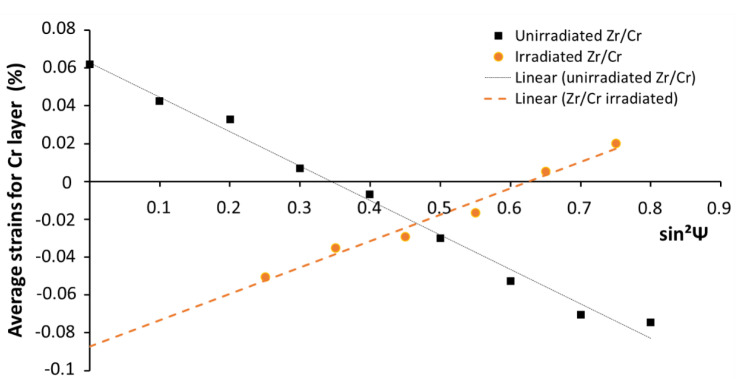
Average strains for Cr layer versus sin^2^Ψ curves for unirradiated (black boxes) and irradiated (orange circles) samples. Dotted lines symbolize the linear regression for the sin^2^Ψ method.

Further, X-ray diffraction performed on the Cr layer before and after ex situ irradiation on a “bulk” Cr-coated sample confirmed that residual compressive stress is present in the as-deposited Cr layer and showed that irradiation contributes to relieve this initial residual stress. Figure 7 shows the evolution of the average strains obtained for the chromium layer versus sin^2^Ψ for both unirradiated and irradiated samples. The stress determination of the as-received (unirradiated) sample highlights a significant compressive stress state (−410 ± 20 MPa). After irradiation, a stress relaxation process occurred and induced a residual tensile stress state (310 ± 20 MPa) at Room Temperature (RT). One can assume that this last tensile stress state should be (at least partially) induced by the cooling from 400 °C down to RT, applied at the end of the irradiation experiments, due to the different expansion coefficients between the Cr and Zr substrate, and thus should not be fully representative of the stress state at 400 °C following the irradiation step.

## 4. Discussion

In this study, we confirmed that the nanometric phase continuous layer can be formed at the Zr–Cr interface after the Cr PVD deposition process. This phase displays a f.c.c. type structure as the C15 ZrCr_2_ equilibrium phase already observed in similar Cr-coated zirconium alloys [7]. However, the lattice parameter is not strictly equal to the expected one, and the chemical composition varies all along with the interface and deviates from the ZrCr_2_ stoichiometry composition. The deviation from the stoichiometry may also result from the fact that the interfacial phase could be a potential stack of nano-sized phases with various composition, as observed by Wu et al. [8]. The relation between lattice parameter and stoichiometric composition has already be proven in other Laves phase systems. Zhu et al. [18] showed that the lattice parameter increases linearly as the Zr content increases in the ZrCo_2_ system. They also reported that the lattice parameters stabilize once the Zr content reached the threshold value of 33%. Therefore, in our case, the deviation of the lattice parameter from the standard value can be linked with the Zr and/or Cr atoms in excess or in deficit, as suggested by the off-stoichiometric composition.

Under in situ ion irradiation, the interfacial phase dissolved, since its width shrunk from 70 ± 10 nm to 6.5 ± 1.4 nm. Further, the dissolution was not accompanied by Zr/Cr atom mixing, as it would have been expected, but rather by a counterintuitive phenomenon: we observed that the interface sharpened rather than broadened. Such a transient interface sharpening has been evidenced experimentally in some various interface systems, such as Mo/V [17], Co-Au [19] and Cu-Ni [20]. However, if sharpening is a well-known phenomenon already observed under thermal annealing [17], to our knowledge it is the first time that this effect is reported under irradiation and will then be discussed in the following. 

By computer simulations, Erdélyi et al. [21] showed that, for strongly composition-dependent diffusion coefficient, an initially diffuse interface of species *A* and *B* can become chemically abrupt even in ideal systems with complete mutual solubility. Erdélyi et al. [17] explained this surprising phenomenon by considering the diffusion coefficient, D. First, in an A-B binary alloy system, diffusion coefficients are often asymmetric, in the sense that one species diffuses faster than the other [20]. Moreover, the atomic jump frequencies for one species may significantly vary with the local composition, leading to composition dependent diffusion coefficients [20]. Thus, for constant *D*, the composition profile will gradually decay and a broadening of the interface is expected. However, when *D* is strongly dependent on the local composition, i.e., *D(C)*, the flux distribution can lead to a sharpening of the interface, as depicted in [17]. Roussel and Bellon [20] explained that for *A-B* binary alloy systems with large asymmetry and with diffusion that decreases as the local *B* concentration increases, the sharpening effect is due to the fact that the tail of the initial *B* composition profile, having a local composition that is almost pure *A*, dissolves rapidly in this *A*-rich phase. This dissolution occurs before any intermixing can take place near the center of the composition profile, leading to a shift of the interface position and to an increase of the composition gradient, i.e., to interface sharpening. Roussel and Bellon [20] brought new insights by showing that sharpening is also dependent to the initial state of the interface and can be suppressed in the case where the interface is initially rough rather than flat and diffuse. In our case, the roughness of the interface appears low, at least at a microscopic scale, and the interface can be considered as “flat and diffuse”.

Wan et al. [22] proposed that sharpening may also be dependent of the presence of vacancy sources or sinks since they observed sharpening of Cu–Ni interfaces only with a high density of sources/sinks. This result is important since irradiation constitutes a significant source of vacancies and the Cr grain boundaries, as well as the Zr–Cr interface and the free surfaces of the FIB lamella, constitute vacancy sinks.

Finally, Erdélyi et al. [21] found that three types of mechanical stresses can also promote the sharpening effect: (i) stress originating from the lattice mismatch at the interface, (ii) thermal stress due to the difference between the thermal expansion coefficient of the layers, and (iii) diffusional stress developing during interdiffusion because of the net volume transport caused by the difference of atomic currents of the constituents through the interface.

In our case, the main difference from the sharpening theory proposed by [17] is that irradiation triggered the sharpening effect instead of thermal annealing. In this paper, radiation-induced sharpening is evidenced but the experimental data collected are not enough to support a clear conclusion on its origin. However, two mechanisms can be proposed as a first approach. 

First, if we consider that following the dissolution of the Zr_x_Cr_y_ phase caused by both ion-beam mixing [23] and low stability due to an off-stoichiometric composition [24], a Zr-Cr solid solution has formed. Then, owing to the low miscibility [25] of these two constituents, it can be imagined that Zr and Cr atoms demix instead of reforming the instable Zr_x_Cr_y_ phase, which leads to the sharpening effect.

**Figure 8:**
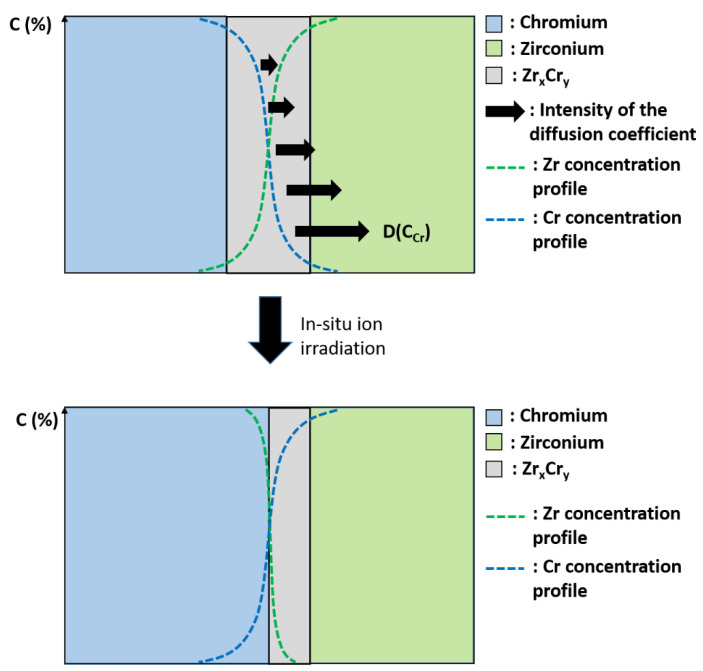
Schematic representation of the sharpening effect caused by asymmetry of the diffusion coefficient.

In a second time, since we are close to the interface condition described by Erdélyi et al. [17], we can imagine a mechanism inspired from the one described in the literature [17,20]. Indeed, residual stress has been found in Zr-Cr systems caused by the mismatch of lattice parameters and the different coefficient of thermal expansion during deposition between coating and substrate [26]. However, if Figure 7 proved that residual stress is well present in the as-deposited Cr layer and that irradiation contributes to relieve it, one has to keep in mind that a sharpening effect was observed on a FIB lamella and not on the bulk sample. It seems reasonable to think that because of their particular low sized geometry, the residual stress of a FIB lamella was relieved during the extraction process. Therefore, the mechanical contribution to sharpening is not obvious in our case. Concerning the vacancy sources/sinks, they exist in the interface region as the free surfaces for instance. The Kirkendall effect has already been observed by Yang et al. [27] in the Zr-Cr system after thermal annealing. They observed the formation of pores at the interface of the Zr-Cr interlayer and the Zr substrate which can be explained by the asymmetry of the diffusion coefficient with Zr diffusing faster than Cr in b.c.c. Cr [28]. Therefore, by considering that the diffusion coefficient depends on the local composition, it can be imagined that Cr atoms will rapidly diffuse and enrich the Zr_x_Cr_y_ phase, causing its destabilization and dissolution. Therefore, the results of Cr enrichment cause the increase of the composition gradient, i.e., the sharpening effect, as illustrated by the abruptness of the chemical profile (Figure 6a) and schematically represented in Figure 8. However, under irradiation, both vacancies and interstitials are created, therefore, radiation-induced segregation might also be taken into account, which can reinforce or suppress the asymmetry of the diffusion coefficient and then lead to a mechanism even more complicated, requiring simulation to be fully understood. However, the fact that the interlayer phase was undissolved after irradiation of the bulk sample [8] may prove that the continuous interlayer Laves-type phase is quite stable under irradiation in the system studied here. Therefore, the dissolution reported in this paper could be well related to the sharpening phenomenon, i.e., the asymmetry of the diffusion coefficient, rather than the well-known radiation-induced dissolution process [23].

Figure 6b presents the composition profile obtained after ex situ Au^5+^ bulk irradiation. This time, contrariwise to the in situ irradiation, the interface broadened instead of sharpened. The same result was also reported by Wu et al. [8] in similar Cr-coated Zircaloy-4 ex situ ion irradiated material. The result discrepancy observed after in situ and ex situ ion irradiation may reside in the fact that sharpening is a transient effect [20,21,22]. Therefore, this may explain why, after ex situ irradiation of a bulk Cr-coated zircaloy-4 sample, broadening of the interface was observed instead of sharpening (Figure 6b). One can imagine that first the interface sharpened and then the interface broadened leading to the decrease of the composition gradient, as illustrated in the chemical profile of Figure 6b. However, another explanation can be proposed. In the case of the irradiation of a bulk sample, the free surface sinks are suppressed. This difference between a bulk sample and FIB lamella may cause the suppression of sharpening, as observed by Wan et al. [21]. Further, as suggested by Figure 1, in the case where the incident ion beam first penetrates the Cr part and then stop into the Zr, the vacancy production is different than in the case where Zr and Cr are irradiated at the same (in situ conditions). This difference may also affect the diffusion coefficient asymmetry and suppress the sharpening effect.

In addition, in this discussion it is worth noting that the behavior of these intermetallics interfacial Zr_x_Cr_y_ phases differs from the behavior of the well-known Secondary Precipitated Phases (SPP). When dispersed within a dilute zirconium based matrix Laves phase, the SPP are not stable under irradiation, but are well known to become amorphous. Gilbert et al. [29] showed that after neutron irradiation at temperatures up to 570 K, Zr(Fe, Cr)_2_ type precipitates exhibit a crystalline core but an amorphous border, while other particles are completely amorphous. Yang et al. [30] also reported that the amorphous transformation of the Zr(Fe, Cr)_2_ under neutron irradiation starts at the periphery of the precipitate and attributed the iron depletion observed as the precipitates becoming amorphous to radiation-induced solute dissolution. Gilbert et al. [29] add that the initial stoichiometry of the precipitates is linked to their ability to become amorphous. Griffiths et al. [31] also observed that the susceptibility of the Zr(Cr, Fe)_2_ particles to the amorphous transformation increases with an increasing Cr content and depends on the depletion of Fe and Cr to below the stoichiometric limit. Further, Griffiths et al. [31] showed that these intermetallic precipitates became amorphous at a low temperature while remaining crystalline at high irradiation temperatures (about 400 °C). Thus, to explain that the Zr_x_Cr_y_ phase formed at the Zr-Cr did not display an amorphous transformation, both after in situ irradiation and ex situ irradiation [8], one may consider that, as shown in this paper, the phases are initially off-stoichiometry and the Cr content could be below the threshold limit reported by Griffiths et al. [31]. Further, the irradiation temperature was 400 °C, that is to say, equal to the temperature where Griffiths et al. [31] observed that precipitates remain crystalline. 

In the end, a brief comment on this transient sharpening effect can be added. One can imagine that such a phenomenon might reorganize the interface structure, which may help in adapting the overall system microstructure to a better response to neutron irradiation. A prospect to this work would be to clarify the effect of the evolution under irradiation of the Cr-coating internal stress state on the sharpening. 

## 5. Conclusions

In this paper, the interface region of Cr-coated Zircaloy-4 materials was studied before and after a specific ion irradiation experiment. In the as deposited Cr-coated sample, a C15 Laves phase-type is observed in the Zr–Cr interface region. This phase possesses the characteristics of the equilibrium ZrCr_2_ Laves phase as it displays an f.c.c structure, but presents a deviation to both stoichiometry composition and reference lattice parameters. During ion-irradiation, in situ experiments showed significant dissolution of this interlayer phase. Further, Energy Dispersion Spectroscopy (EDS) revealed that the phase dissolution is accompanied by an increase of the concentration profile abruptness, which corresponds to a counterintuitive effect, i.e., radiation-induced sharpening phenomenon. However, under ex situ irradiation of “bulk” samples, a significant evolution of the Cr-coating internal stress state is observed and the Zr-Cr profile appears broad instead of sharp, which tends to prove that the sharpening effect should be transient and potentially related to the specific effects of irradiation of a thin (foil) sample. Then, further additional experimental and modeling efforts should be done to better support the observed Zr–Cr interface evolution under ion irradiation, and, finally, to extend such analysis to neutron irradiation. 

## Figures and Tables

**Table 1:** Chemical composition (wt.%) of the Zircaloy-4 (Zy-4).

Sn	Cr	Fe	O	Zr
1.2–1.7	0.1	0.18–0.24	0.1–0.14	Bal.

## Data Availability

The data presented in this study are available on request from the corresponding author. The data are not publicly available due to privacy property.
